# Feasibility and Acceptability of a Combined Digital Platform and Community Health Worker Intervention for Patients With Heart Failure: Single-Arm Pilot Study

**DOI:** 10.2196/47818

**Published:** 2023-10-02

**Authors:** Jocelyn Carter, Natalia Swack, Eric Isselbacher, Karen Donelan, Anne N Thorndike

**Affiliations:** 1 Division of General Internal Medicine Massachusetts General Hospital Harvard Medical School Boston, MA United States; 2 Division of General Internal Medicine Massachusetts General Hospital Boston, MA United States; 3 Corrigan Minehan Heart Center Massachusetts General Hospital Harvard Medical School Boston, MA United States; 4 Heller School for Social Policy and Management Brandeis University Waltham, MA United States

**Keywords:** heart failure, digital platform, remote monitoring, home-based care, community health worker, social needs care, community health work, care, monitoring, pilot study, heart, feasibility, acceptability, community, heart rate, oxygenation, willingness, mobile phone

## Abstract

**Background:**

Heart failure (HF) is one of the leading causes of hospital admissions. Clinical (eg, complex comorbidities and low ejection fraction) and social needs factors (eg, access to transportation, food security, and housing security) have both contributed to hospitalizations, emphasizing the importance of increased clinical and social needs support at home. Digital platforms designed for remote monitoring of HF can improve clinical outcomes, but their effectiveness has been limited by patient barriers such as lack of familiarity with technology and unmet social care needs. To address these barriers, this study explored combining a digital platform with community health worker (CHW) social needs care for patients with HF.

**Objective:**

We aim to determine the feasibility and acceptability of an intervention combining digital platform use and CHW social needs care for patients with HF.

**Methods:**

Adults (aged ≥18 years) with HF receiving care at a single health care institution and with a history of hospital admission in the previous 12 months were enrolled in a single-arm pilot study from July to November 2021 (N=14). The 30-day intervention used a digital platform within a mobile app that included symptom questionnaire and educational videos connected to a biometric sensor (tracking heart rate, oxygenation, and steps taken), a digital weight scale, and a digital blood pressure monitor. All patients were paired with a CHW who had access to the digital platform data. A CHW provided routine phone calls to patients throughout the study period to discuss their biometric data and to address barriers to any social needs. Feasibility outcomes were patient use of the platform and engagement with the CHW. The acceptability outcome was patient willingness to use the intervention again.

**Results:**

Participants (N=14) were 67.7 (SD 11.7) years old; 8 (57.1%) were women, and 7 (50%) were insured by Medicare. Participants wore the sensor for 82.2% (n=24.66) of study days with an average of 13.5 (SD 2.1) hours per day. Participants used the digital blood pressure monitor and digital weight scale for an average of 1.2 (SD 0.17) times per day and 1.1 (SD 0.12) times per day, respectively. All participants completed the symptom questionnaire on at least 71% (n=21.3) of study days; 11 (78.6%) participants had ≥3 CHW interactions, and 11 (78.6%) indicated that if given the opportunity, they would use the platform again in the future. Exit interviews found that despite some platform “glitches,” participants generally found the remote monitoring platform to be “helpful” and “motivating.”

**Conclusions:**

A novel intervention combining a digital platform with CHW social needs care for patients with HF was feasible and acceptable. The majority of participants were engaged throughout the study and indicated their willingness to use the intervention again. A future clinical trial is needed to determine the effectiveness of this intervention.

## Introduction

Heart failure (HF) is one of the most common causes of hospitalization and generates significant expense and hardship for patients [1,2]. Numerous adverse clinical (eg, low ejection fraction, elevated natriuretic peptide, and complex disease comorbidities) [3,4] and social needs care factors (eg, transportation, food security, housing stability, and access to care) [5,6] have been associated with HF exacerbations leading to hospitalization along with rising cost of HF care [7]. Growing pressure to provide more effective clinical support to patients with HF at home and reduce total costs of care has resulted in increased interest in biometric monitoring with digital solutions paired with wearable devices that can track data for patients and care teams [8]. Similarly, home-based interventions addressing social needs care from a navigator or community health worker (CHW) have shown promise addressing gaps in social needs care while impacting clinical outcomes in chronic disease populations [9,10].

Digital platforms can offer a suite of remote biometric monitoring such as continuous heart rate and oxygenation monitoring, and measurement of blood pressure and body weight via digital devices [11-13]. Machine learning algorithms may also contribute to framework accuracy and alert specificity [14]. Use of digital platforms to determine early clinical decline (eg, changes in baseline weight, activity tolerance, heart rates, and blood pressure) via biometric and symptom-linked monitoring has the potential to benefit HF home care. However, broad adoption and implementation of digital platforms for patients with HF have lagged for a variety of reasons stemming from institutional and provider-related challenges to logistical constraints in tech-challenged settings and aging populations [15]. Even in settings that are able to support digital solutions, there are patient barriers to digital platform use such as knowledge gaps, lack of willingness to adopt new skills, reluctance to use technologies, and challenges with internet connectivity and access [16,17]. As a result, concerns about the exclusion of digital platform use in low-resourced or less technology savvy populations persist [18]. A number of these identified patient barriers could be addressed by integrating a home-based human resource with the capacity to guide patients through platform use while also addressing any unmet social needs care.

As lay professionals trained with basic knowledge of chronic conditions, such as HF, home-based CHW staff spend time with patients, often addressing social care needs [6] (eg, economic, educational, and behavioral factors) that influence clinical outcomes such as unplanned hospital readmissions and emergency department (ED) visits. These activities could include supporting patients in the logistics of navigating digital platform use and connectivity as it relates to home monitoring [19]. As such, home-based CHW roles [9,10,20,21] are uniquely positioned to support digital platform use and engagement by also supporting patients with any needed resources such as connectivity, transportation, nutrition education, or psychosocial support [22].

In this study, we explore a combined remote monitoring and CHW intervention designed for patients with HF and unmet social needs who had at least 1 hospitalization in the prior 12 months. We conducted a single arm pilot study to determine the feasibility and acceptability of this intervention as preliminary data for a larger clinical trial.

## Methods

### Setting and Study Design

We enrolled 14 participants who had a diagnosis of HF (July 2021 through November 2021) and at least 1 hospitalization in the prior 12 months. A 15th participant was initially enrolled but later excluded due to the inability to sustain connectivity of the digital platform at home despite the trial use of multiple sets of platform hardware; this was thought to be due to interference related to connectivity of other devices within the home. Study participants were identified using 6 outpatient primary care and cardiology clinic panels of clinicians at Massachusetts General Hospital (MGH). MGH is a 999-bed academic medical center in Massachusetts. Further, 15 MGH primary care clinics and the MGH Corrigan Minehan Heart Center support over 27,000 cardiac patients each year; many of whom have a HF diagnosis. A HF diagnosis was defined as having this condition (systolic or diastolic HF) listed in the electronic health record (EHR) problem list.

### Ethical Considerations

Institutional review board approval was obtained from the Massachusetts General Brigham Human Research Committee on September 22, 2020 (2018P002014). All enrolled participants provided written consent for study enrollment. It was given prior to this study. All methods were carried out in accordance with guidelines and regulations outlined by the Mass General Brigham institutional review board. All participants were provided US $250 of remuneration for study participation.

### Participants

Eligibility criteria were developed based on findings from prior qualitative studies focused on perspectives on hospital to home care transitions and home-based care that includes HF orts [23-25]. Specifically, in prior interviews with patients with HF who had a history of prior hospitalization, themes related to difficulty adhering to diet and medication instructions outlined by primary care or cardiology specialists and willingness to participate in digital platform interventions emerged. Based on this, study participants with HF were recruited from outpatient cardiology and internal medicine clinic panels. Participants had to be ≥18 years old, live within a 30-mile radius of MGH, have a diagnosis of HF listed in the EHR problem list, have a history of ≥1 hospitalizations within the previous 12 months, a clinician managing their HF, smartphone use familiarity, ability, and willingness to participate in the intervention, and English fluency. Patients were ineligible if they were experiencing homelessness, had an active alcohol or substance use disorder, were living in a long-term care facility, were unable to provide consent due to cognitive impairment, or had invoked health care proxy or prisoner status.

### Enrollment

Research staff called patients expressing prior interest in study involvement by phone to describe the study, establish eligibility, and enroll participants. Patients were called up to 3 times if they were unsure or unable to be contacted on initial outreach. If interested, patients agreed to meet study staff at MGH main campus for enrollment and were enrolled after consent processes were completed. Enrolled participants were introduced to the digital platform (Biofourmis) [26] features: a HF mobile phone app on an Android phone that included a daily checklist of patient to-do items, educational HF videos, a portal for messaging the assigned CHW, and a daily symptom questionnaire (see Multimedia Appendix 1). In addition, participants were provided with a digital blood pressure monitor, a digital weight scale, and a sensor attached to a lightweight arm band worn on the nondominant arm tracking basic biometrics (heart rates, oxygenation, and steps taken; see Multimedia Appendix 2). The digital platform was designed for patients with HF by the platform developers and specific aspects of the platform dashboard layout and alert deployment were modified for CHW use by platform developers prior to study start. These modifications were led by a CHW staff member trained to guide patients in navigation of the platform as well as members of the research team (eg, JC and NS). The CHW contacted participants via phone within 24 weekday hours of enrollment.

### Intervention Care

Participants were given access to the digital platform, BiofourmisRPM, along with instruction on the use of all devices for the 30-day intervention. Participants were encouraged to wear the sensor via armband for up to 24 hours daily and check blood pressure and weight daily. Participants were encouraged to complete a daily symptom questionnaire and view American Heart Association HF educational videos using the mobile app. Baseline biometrics were established by participant use of the digital platform on hospital discharge (whereupon baseline heart rates, oxygenation rates, and steps taken along with blood pressures and body weights were established). A machine learning algorithm within the mobile app generated a daily Biovitals Index for the CHW team dashboard with alerts sent to the CHW team dashboard indicating if participants were at, moving away from, or moving toward their clinical baseline in terms of symptoms, biometrics, and functionality. The team also applied a color schematic, red (recommendation for immediate clinical attention; score ≥1.0), yellow (recommendation for clinical attention within 24 hours; score 0.8-0.9), green schematic (no clinical attention required; score 0.7), which was used to further categorize patient status and needed actionability. No mobile app-generated feedback was given to patients based on abnormal biometrics or symptoms.

Specifically, CHWs were trained to assess scores and alerts generated by the digital platform. Any scores or alerts indicating that participants were moving away from their baseline were discussed with a CHW project manager (ie, trained intensive care unit nurse). When indicated, the CHW staff connected with patients whose biometric data or symptoms were within the yellow or red zones within 4 workday hours of occurrence. Patients were instructed to use on-call clinical team or urgent or emergent care as they normally would if they developed new symptoms or concerning changes in biometric measurements after workday hours or on weekends. The CHW was also trained to assist patients with the technology set up and trouble shooting. Any unreconciled technical difficulties were addressed by research study staff and the platform vendor, as needed.

Participants received routine calls and contacts from their assigned CHW to address barriers to social needs care as well as patient knowledge gaps or challenges with adherence to clinical care plans. Social care needs included any issues related to transportation, insurance benefits, food security, rental, or housing assistance (or any unmet need related to the social determinants of health), as well as psychosocial support. Further, 1 CHW with expertise in CHW core competencies [27] (motivational interviewing, goal-setting, behavior change, and psychosocial support) delivered the intervention and was trained to assist participants with the digital platform. The CHW documented all encounters in the EHR (eg, enrollment notes, progress notes for all contacts) as well as completing the CHW interaction by logging in a REDCap database. Preexisting clinical team members were copied on all EHR notes and contacted directly by the CHW or supervisory staff during the intervention when needed.

In total, 1 CHW staff member was involved in this intervention as a hospital employee. This CHW completed extensive training in self-management as part of an 80-hour course associated with CHW certification supported by the local public health commission. Focused case-based learning specific to the digital platform use and HF home care led by the principal investigator (JC) and a CHW supervisor, clinically trained as a critical care nurse, was conducted prior to the study. Training on the digital platform and HF care occurred over a 2 week period (6 hours in total) culminating with skill-based proficiency assessments.

### Measures or Outcomes

All study participants completed an enrollment questionnaire to establish baseline habits and patient experience adapted from previous survey instruments [23,24]. This questionnaire was derived from standard established measures of patient experience for benchmarking as well as validated questions generated by prestudy qualitative interviews with patients and discussions with physicians caring for patients with HF. The enrollment questionnaire included items about health-related habits, understanding of the care plan, smartphone knowledge, quality of life, perceptions of physical and mental health, unmet social needs, loneliness, and depression. At study end, participants completed an exit interview focused on their experiences with the technology and an acceptability questionnaire focused on the digital platform and their experiences with the CHW. For the usability and acceptability interview questions, components of the System Usability Scale [28], Post Study System Usability Questionnaire [29], Technology Assessment Model Measurement Scales [30], and Usefulness, Satisfaction, and Ease Questionnaire [31] were adapted as described by Ben-Zeev et al [32] for the purposes of this study. For the acceptability questionnaire, the responses were “very true,” “somewhat true,” and “not true.” For the CHW experience, responses ranged on a scale from “satisfied” to “neutral” to “not satisfied.” The questionnaires were initially pretested with 3 patients and no additional changes were made. The exit interview and all questionnaires were verbally administered by study staff. Basic demographics, insurance status, and major medical and psychiatric comorbidities were collected via EHR chart review. The primary outcomes were feasibility and acceptability of the combined digital remote monitoring and CHW intervention designed for patients with HF. Feasibility outcome measures included daily use rates of the biometric sensor (average hours per day), the digital blood pressure monitor (average times per day), the weight scale (average times per day), and completion of the symptoms questionnaire (average times per day). The acceptability outcome measure was captured by patients’ response to the statement that if given the opportunity, they would use the digital platform again in the future (response options: very true, somewhat true, or not true).

Demographic data and survey item responses were captured in REDCap (Research Electronic Data Capture; Vanderbilt University). These items were summarized, and univariate analysis was completed for any domains connected to the outcomes. Structured medical record review data extracted from the EHR were also captured in a REDCap database.

### Statistical Analysis

Univariate analysis included demographic covariates of participants and intervention use frequencies, means, and SDs related to feasibility and acceptability outcomes. A brief 4-item qualitative exit interview guide probing the participant experience was administered at the end of the study and framework analysis was used to identify main themes [33]. We conducted coding and analysis using verbatim transcription. The transcripts were uploaded into Dedoose (software version 8.3.47B.exe; SocioCultural Research Consultants, LLC) for analysis.

## Results

### Overview

In total, 14 participants with HF were enrolled and included in the analysis (Table 1) in this observational study between July and November 2021. Participants (n=14) were 68.7 (SD 11.7) years old; 8 (57.1%) were women, and 7 (50%) were insured by Medicare. Of those enrolled, 6 (42.8%) had HF with a reduced ejection fraction (≤40%; Table 1). All participants completed the enrollment and exit questionnaires or interview.

**Table 1:** Patient characteristics (N=14).

Participant characteristics			Value
Female sex, n (%)			8 (57.1)
Age (years), mean (SD)			68.7 (11.7)
**Race and ethnicity, n (%)**			
	Asian, non-Hispanic	0 (0)	
	Black, non-Hispanic	1 (7.1)	
	Hispanic or Latin	0 (0)	
	White, non-Hispanic	13 (92.9)	
**Primary insurance, n (%)**			
	Medicare	7 (50)	
	Commercial or private	6 (42.9)	
	Medicaid	1 (7.1)	
**Highest level of education, n (%)**			
	≤High school	3 (21.4)	
	≥Some college or more	11 (78.6)	
**Heart failure, n (%)**			
	Ejection fraction <40%	6 (42.8)	
	Ejection fraction ≥40%	8 (57.1)	
**Medical history, n (%)**			
	Hypertension	11 (78.6)	
	Hyperlipidemia	6 (42.9)	
	Atrial fibrillation	5 (35.7)	
	Diabetes	5 (35.7)	
	Chronic kidney disease	5 (35.7)	
	Non-ST elevation myocardial infarction	4 (14.8)	
	≥2 or more chronic conditions	14 (100)	
**Usage or needs, mean (SD)**			
	Hospitalizations in 12 months prior to enrollment	1.2 (0.47)	

### Feasibility

**Figure 1:**
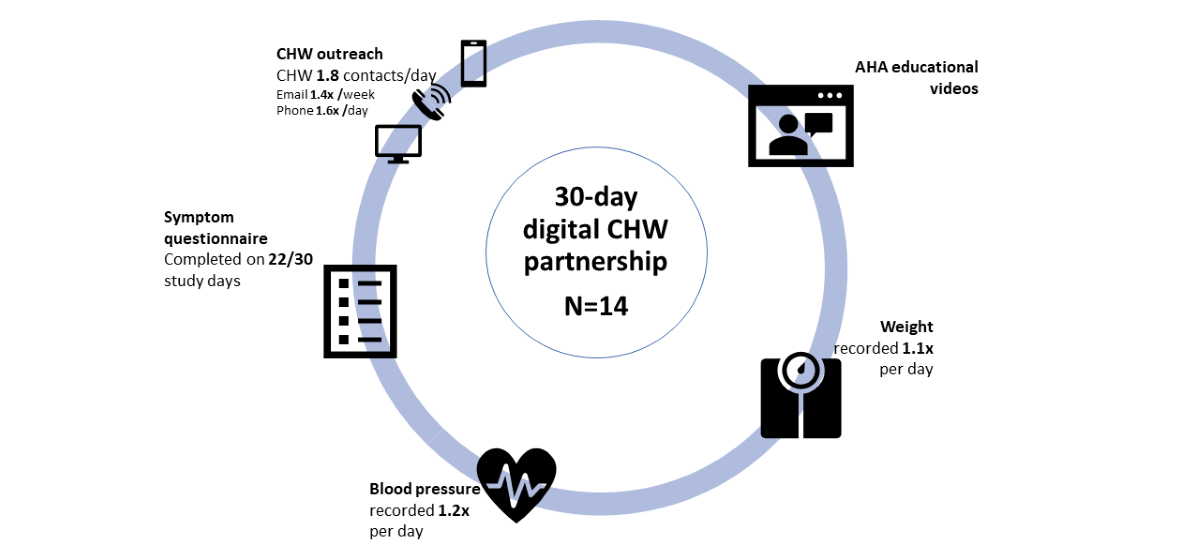
Description of Biofourmis mobile app function for 14 study participants over a 30-day study period. The mobile app included educational videos and articles from the American Heart Association, the ability to record weight and blood pressure, and a daily 4-question symptom questionnaire. AHA: American Heart Association; CHW: community health worker.

All participants used the digital platform throughout the 30-day study interval and wore the sensor for 82.2% (n=24.66) of study days with an average of 13.5 (SD 2.1) hours per day (Figure 1). Participants used the digital blood pressure monitor and digital weight scale at a mean of 1.2 (SD 0.17) times per day and 1.1 (SD 0.1) times per day, respectively. All participants completed the symptom questionnaire on at least 71% (n=21.3) of study days.

Overall, 11 (78.6%) of participants had ≥3 CHW interactions (Table 2), and all participants had at least 2 CHW interactions. Of the 311 total patient-CHW interactions occurring during the study interval, the most frequent CHW activities were related to reinforcement of the general care plan (n=113, 36.3%), psychosocial support (n=45, 14.5%), salt or nutrition education (n=40, 12.9%), making clinical appointments (n=22, 7.5%), case management referrals (n=18, 5.8%), and creating reliable transportation (n=14, 4.5%).

**Table 2:** Types of community health worker-patient interactions (N=311).

Interactions		Value, n (%)
**Medical needs**		
	Reinforcement of general adherence	113 (36.3)
	Making or confirming clinical appointments	22 (7.1)
	Assessment of need of case management services	18 (5.8)
	Assisted patient with insurance or eligibility inquiries	10 (3.2)
	Referral to a health agency	1 (0.3)
	Arranging for access to medications	2 (0.6)
	Other	1 (0.3)
**Coaching or teaching**		
	Psychosocial support	45 (14.5)
	Education on salt, fluid, or other aspects of dietary intake or nutrition	40 (12.9)
	Education on activity	11 (3.5)
	Education on medications	7 (2.3)
	Education on other aspects of general health	4 (1.3)
**Social or basic needs**		
	Transportation for clinical care	14 (4.5)
	Referral to a social service agency	8 (2.6)
	Referral to social work	7 (2.3)
	Assistance with basic needs (eg, housing, food, and electricity)	6 (1.9)
	Financial counseling or eligibility for program assistance	2 (0.6)

### Acceptability

All participants completed exit questionnaires. Overall, 10 (71.4%) participants responded that the statement “I found that the different parts of the [digital platform] worked well together” was very true or somewhat true. Furthermore, 11 (78.6%) participants indicated that the statement “If I have access to the [digital platform] moving forward, I will use it” was very true or somewhat true, and 9 (64%) indicated that the statement “I think I would need the support of a technical person” to use the digital platform was very true or somewhat true. In total, 12 (86%) participants indicated that their CHW interaction was satisfying.

Four themes emerged from patient exit interviews (Textbox 1): (1) patients with HF value access to data via remote monitoring at home, (2) participants experienced inconsistencies with the accuracy and precision of the digital platform, (3) patients viewed the CHW role as beneficial in terms of motivation, connection to resources, and assistance with insurance, and (4) patients with HF see digital platform use as generally helpful with little inconveniences.

**Major themes associated with illustrative quotes from poststudy interviews with participants with heart failure.**

- Data: *patients with heart failure value access to data via remote monitoring at home*
  “Well, I think that it's a positive thing to have it in the home. It gives you a lot of information, and it's not intimidating.”
  “And the cuff thing, I found out a lot of information. Because I have CPAP, that I hate.”
  “[In the past] with my doctor.. I was forwarding her exactly what was happening with you guys’ and technology, but I was doing it by taking a picture and sending it to her through the Gateway. So it was a little archaic now that I've done this. It seems very archaic and it would have been simple …if this technology was available, it really would have been a lovely thing because I did it twice a day before..”
  “Okay. I found it okay. I mean, watching my weight, that was great. Watching my blood pressure was even better, because I [have] problems.”
  “Yeah. I mean….it was extremely informative. It was helpful. It gave your blood pressure. It gave your oxygen level. It gave your skin temperature.”
- Digital issues: *participants experienced inconsistencies with the accuracy and precision of the digital platform.*
  “I mean, my device had to be restarted every so often which was inconvenient.”
  “[The digital scale] had me from 176 to 224 to 233 back down to 170s-- so it was all over the place one day.”
  “I mean, if it was not for the few glitches, then I would say that it works perfectly for me.”
- Community health worker (CHW): *patients viewed the CHW role as beneficial in terms of motivation, connection to resources, and assistance with insurance.*
  “I think that [the CHW] motivated me to follow up with my senior center … because there seems to be a number of classes that I could take.”
  “Like I say, I really appreciated that [the CHW] sort of walked me, held my hand as we went through a couple of the application processes. Yeah, it was very good.”
  “[The CHW] was excellent. She helped me with some upcoming things that I have to deal with, Medicare specifically. There's some other things I had asked [the CHW] help about, right away, [the CHW] obtained the information for me and shared it with me, the resources. Yeah, [the CHW] was very good.”
- Platform use: *patients with heart failure see digital platform use as generally helpful with little inconveniences.*
  “So the device, I honestly didn't really notice much of a difference in my normal life, except something obviously on my arm. But that really wasn't a problem for me. I normally take my blood pressure often and my weight often. So that wasn't anything new to me, except to record it. So that really wasn't a problem.”
  “The phone that I used was different from an iPhone, but it came up easy enough for me to find the menus and to do what I needed to do on them..”
  “It was great. I loved it. You know what I mean.”

### Exploratory Findings

**Figure 2:**
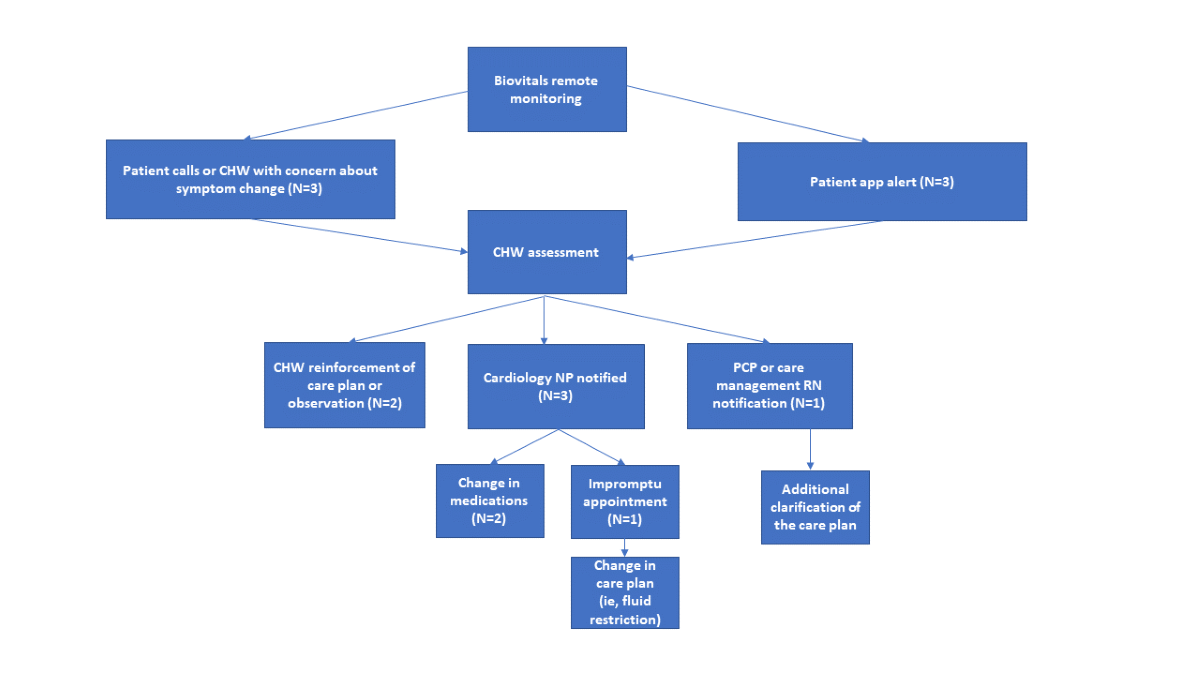
Examples of care team notification of patient decline. Occurrences were initiated due to a change in patient symptoms identified by machine learning algorithm Biovitals alerts. Both a change in patient symptoms reported to their CHW via phone and generated Biovitals dashboard alerts from remote monitoring resulted in CHW assessment. From there, the appropriate steps were taken as identified by the study team. This often resulted in CHW reinforcement of care, notification of the patient’s cardiology team, or notification of the patient’s primary care team which lead to medication changes, impromptu appointments, care plan changes, and care plan clarification. CHW: community health worker; NP: nurse practitioner; PCP: primary care physician.

Exploratory analysis also demonstrated that there were 6 occurrences where the intervention may have assisted in preventing clinical decline, ED visits, or hospital admissions (Figure 2). These occurrences were identified by biometric changes generated by the digital platform as well as patient symptoms spurring CHW assessment. Further, 4 of these occurrences resulted in care team communications, encounters, and changes in clinical care plans. These changes potentially addressed factors that may have otherwise resulted in ED visits or hospital readmissions. Examples of how clinical teams were notified of patient decline are described in detail in Figure 2.

## Discussion

### Principal Findings

In this single arm study assessing the feasibility and acceptability of digital platform use combined with CHW care for patients with HF, we found that the intervention was both feasible and acceptable. The digital platform was designed for patients with HF and modified for use by a CHW staff member trained to guide patients in navigation of the platform. This patient-centered and comprehensive home-based HF management intervention was both feasible and acceptable to patients.

Prior studies have demonstrated the willingness of patients with HF to engage with digital platforms, and this was consistent with our findings [34]. Although the biometrics of the digital platform such as heart rate, steps taken, oxygenation, and blood pressure were generally accurate, qualitative interviews suggested that some participants initially experienced weight scale errors due to an incomplete mobile app update that was subsequently corrected. This finding underlines the importance for measurement accuracy and precision in home-based HF interventions where clinical status often hinges on narrow margins of change in weight or other biometrics [35,36]. The effect of care plan reinforcement or other categories of CHW-patient interactions seen here in this study have not been well studied in digital remote monitoring interventions. However, the impact of these types of CHW activities is strongly supported in prior general medicine and disease-specific CHW studies [12,37,38].

There were some unexpected findings in this study. First, most participants indicated in the poststudy questionnaire that they would need in-person support in order to use the digital platform. This finding is supported in at least 1 prior observational study [32] and reflects patient perceptions about the technological challenges of digital platform use in this older and chronically ill population of patients. Most participants indicated in the poststudy questionnaire that they “needed to learn a lot of things before using the digital platform.” This supports the use of embedding assistance in the form of a CHW as a necessary component of digital platform use and engagement [33,39].

In exit interviews, participants also identified aspects of the digital platform that could be improved. These included improving weight scale precision and accuracy, optimizing digital platform connectivity, and streamlining the mobile app layout. Addressing these participant-identified areas of improvement may avoid patient-perceived logistical and technical inconveniences associated with using the digital platform and further enhance patient engagement and intervention adoption.

We identified several examples where the intervention resulted in additional CHW assessment, clinical care team coordination, or care plan changes without resulting in acute care use or hospitalization. These examples, triggered by patient symptoms or digital platform alerts, resulted in continued observation, medication changes, or an impromptu appointment. Subsequent involvement of the patient clinical home occurred on a case-by-case basis. Future study of these pathways can identify additional opportunities for intervention and determine if the intervention helps to prevent emergency visits or hospitalizations.

### Limitations

Since this was an observational single-arm study, we cannot establish effectiveness of the intervention. Furthermore, our sample size was small, mostly White, and limited to a single academic institution; these results may not be generalizable to other settings. Despite this, we believe that the novel intervention and use of patient response data gathered from questionnaires and exit interviews provides meaningful context for the acceptability and feasibility of the intervention. Furthermore, given the timing of the study with performance during the height of the COVID-19 pandemic, enrollment in the outpatient setting may have been affected by patients who were isolating in their homes versus those who were unable to be contacted due to living elsewhere at that time.

### Conclusions

Use of a digital platform combined with a CHW is both feasible and acceptable for delivering home-based HF care. Given the significant burden for patients with HF living at home, this intervention could help predict early decline, guide intervention prior to clinical deterioration, and prevent hospital admissions and ED visits. A future randomized controlled trial is needed to test the preliminary effectiveness of this intervention in improving clinical outcomes.

## Appendix

Multimedia Appendix 1 Digital platform symptom questionnaire.

Multimedia Appendix 2 Digital platform biosensor on wristband.

## Abbreviations

CHW: community health worker
ED: emergency department
EHR: electronic health record
HF: heart failure
MGH: Massachusetts General Hospital
REDCap: Research Electronic Data Capture

## Data Availability

Deidentified data may be obtained upon request by contacting the corresponding author with a descriptive proposal stating the purpose of the data request.
