# Supplementary material for: Feasibility and Acceptability of a Combined Digital Platform and Community Health Worker Intervention for Patients With Heart Failure: Single-Arm Pilot Study
Source: JMIR Cardio. 2023 Oct 2;7:e47818. doi: 10.2196/47818 (PMC10580132; doi:10.2196/47818)
Supplement: Multimedia Appendix 1 [file cardio_v7i1e47818_app1.docx]

Patient Daily Questionnaire

1. Which of the following best describes your breathing today?
   1. No new or worsening shortness of breath
   2. Some worsening shortness of breath with activity
   3. Some shortness of breath at rest
   4. Unsure
2. Which of the following best describes your swelling today?
   1. No new swelling; feet and legs look normal to you
   2. Increased swelling of legs, feet, or ankles
   3. Unsure
3. Which of the following best describes you today?
   1. No new chest pain symptoms
   2. New chest pain symptoms
   3. New chest heaviness
   4. Unsure
4. Which of the following describes your weight today?
   1. No weight change
   2. Weight gain of more than 2-3 lbs in the last 24 hours
   3. Weight gain of more than 5 lbs in the last week
   4. Unsure
