# Supplementary material for: Feasibility and Acceptability of a Combined Digital Platform and Community Health Worker Intervention for Patients With Heart Failure: Single-Arm Pilot Study
Source: JMIR Cardio. 2023 Oct 2;7:e47818. doi: 10.2196/47818 (PMC10580132; doi:10.2196/47818)
Supplement: Multimedia Appendix 2 [file cardio_v7i1e47818_app2.docx]

Biofourmis wireless biometric sensor and armband


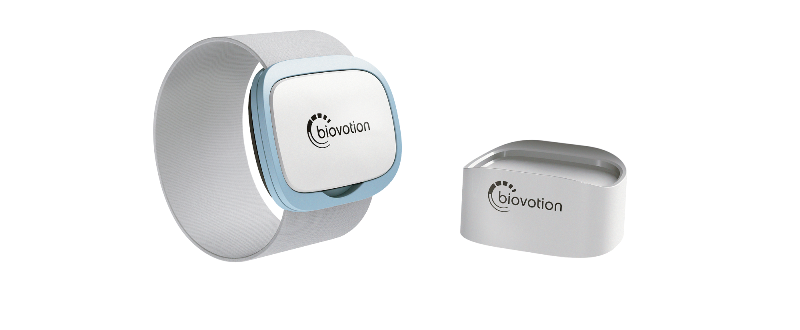

Medical Device Network (2019, November 19) *Biofourmis signs agreement to acquire Biovotion. https://www.medicaldevice-network.com/news/biofourmis-acquires-biovotion/?cf-view*
